# Oral Contraceptive Use Influences On-Kinetic Adaptations to Sprint Interval Training in Recreationally-Active Women

**DOI:** 10.3389/fphys.2020.00629

**Published:** 2020-06-12

**Authors:** Mia Annalies Schaumberg, Jamie Stanley, David G. Jenkins, Emily A. Hume, Xanne A. K. Janse de Jonge, Lynne M. Emmerton, Tina L. Skinner

**Affiliations:** ^1^School of Health and Sport Sciences, University of the Sunshine Coast, Sippy Downs, QLD, Australia; ^2^Sunshine Coast Health Institute, Birtinya, QLD, Australia; ^3^School of Human Movement and Nutrition Sciences, The University of Queensland, Brisbane, QLD, Australia; ^4^South Australian Sports Institute, Kidman Park, SA, Australia; ^5^Australian Cycling Team, Gepps Cross, SA, Australia; ^6^Allied Health and Human Performance, University of South Australia, Adelaide, SA, Australia; ^7^School of Clinical Medicine, The University of Queensland, Brisbane, QLD, Australia; ^8^School of Environmental and Life Sciences, The University of Newcastle, Ourimbah, NSW, Australia; ^9^School of Pharmacy and Biomedical Sciences, Curtin University, Perth, WA, Australia

**Keywords:** female, training adaptation, ovarian hormones, oral contraceptive (OC), exogenous hormones, cardiorespiratory, time-to-fatigue

## Abstract

**Introduction:**

Oral contraceptive (OC) use influences peak exercise responses to training, however, the influence of OC on central and peripheral adaptations to exercise training are unknown. This study investigated the influence of OC use on changes in time-to-fatigue, pulmonary oxygen uptake, cardiac output, and heart rate on-kinetics, as well as tissue saturation index to 4 weeks of sprint interval training in recreationally active women.

**Methods:**

Women taking an oral contraceptive (OC; *n* = 25) or experiencing natural menstrual cycles (MC; *n* = 22) completed an incremental exercise test to volitional exhaustion followed by a square-wave step-transition protocol to moderate (90% of power output at ventilatory threshold) and high intensity (Δ50% of power output at ventilatory threshold) exercise on two separate occasions. Time-to-fatigue, pulmonary oxygen uptake on-kinetics, cardiac output, and heart rate on-kinetics, and tissue saturation index responses were assessed prior to, and following 12 sessions of sprint interval training (10 min × 1 min efforts at 100–120% PPO in a 1:2 work:rest ratio) completed over 4 weeks.

**Results:**

Time-to-fatigue increased in both groups following training (*p* < 0.001), with no difference between groups. All cardiovascular on-kinetic parameters improved to the same extent following training in both groups. Greater improvements in pulmonary oxygen up-take kinetics were seen at both intensities in the MC group (*p* < 0.05 from pre-training) but were blunted in the OC group (*p* > 0.05 from pre-training). In contrast, changes in tissue saturation index were greater in the OC group at both intensities (*p* < 0.05); with the MC group showing no changes at either intensity.

**Discussion:**

Oral contraceptive use may reduce central adaptations to sprint interval training in women without influencing improvements in exercise performance - potentially due to greater peripheral adaptation. This may be due to the influence of exogenous oestradiol and progestogen on cardiovascular function and skeletal muscle blood flow. Further investigation into female-specific influences on training adaptation and exercise performance is warranted.

## Introduction

Exogenous hormones introduced through oral contraceptive (OC) use can reduce maximal exercise capacity (Notelovitz, 1987; Casazza et al., 2002; Lebrun et al., 2003), increase fat-mass (Berenson and Rahman, 2009) and change the metabolic (Isacco et al., 2012), thermoregulatory (Stachenfeld et al., 2000), cardiovascular (Coney et al., 2001), and ventilatory (Charkoudian and Joyner, 2004) responses to exercise. Although OC use has been shown to impede peak exercise adaptations to training (Schaumberg et al., 2017a), the physiological mechanisms and whether performance adaptations are influenced by OC use is unclear (Casazza et al., 2002; Lebrun et al., 2003; Zierke, 2010).

One potential mechanism mediating training adaptation is skeletal muscle blood flow. During incremental exercise, skeletal muscle blood flow is determined by locally induced vasodilation and sympathetically mediated vasoconstriction, both of which are influenced by ovarian hormones (Charkoudian and Johnson, 1997; Charkoudian and Joyner, 2004; Vanheest et al., 2005). Indeed, chronic estrogen exposure has known vasodilatory responses (Stathokostas et al., 2009), and there is some evidence to suggest that estrogen and progestogen supplementation and the menstrual cycle may influence blood flow during exercise (Gurd et al., 2007), the directionality of which appears to depend on multiple factors, including the phase of the menstrual cycle (i.e., the ratio of estrogen and progesterone) and the type and concentration of exogenous ovarian hormone concentrations. Altered muscle blood flow due to endogenous and exogenous ovarian hormones may influence the ability of the muscle to meet oxidative demands during exercise. However, this has not been investigated, nor has OC use been considered as a potential mediator of skeletal muscle blood flow adaptations to exercise training in women.

A further potential mechanism is the integration of pulmonary and cardiovascular systems to deliver oxygenated blood to skeletal muscle during exercise. Pulmonary oxygen uptake on-kinetics (τV̇O_2_*_*p*_*) provide insight into how the cardiovascular system and mitochondria integrate to increase aerobic energy production in response to exercise (Murias et al., 2011). The speed of τV̇O_2_*_*p*_* is a good indicator of endurance performance (Jones and Burnley, 2009); a faster τV̇O_2_*_*p*_* indicates earlier achievement of physiological steady state (indicated by the time constant, τ*on*, or the mean response time, MRT) resulting in reduced oxygen deficit (Xu and Rhodes, 1999). Additionally, a fast τV̇O_2_*_*p*_* has also been associated with reduced lactate accumulation and muscle glycogen depletion compared to a slow τV̇O_2_*_*p*_* (Berger et al., 2006). There is increasing interest in optimal training methods to elicit physiological adaptations within pulmonary, cardiovascular, and muscular systems to improve exercise performance.

Sprint interval training (SIT) can elicit adaptations traditionally associated with endurance training in a shorter period (Gibala et al., 2012; Shiraev and Barclay, 2012). These adaptations appear to be independent of sex and include improved oxidative enzyme activity (Carter et al., 2001; Astorino et al., 2011) coupled with increased capillarization (Shiraev and Barclay, 2012) and more efficient blood distribution (Murias et al., 2011), which can lead to improvements in V̇O_2__peak_. These adaptations also improve the rate at which oxygen is extracted in the lungs (τV̇O_2__p_) (Shoemaker et al., 1996; Tschakovsky and Hughson, 1999; Grassi, 2001; Hughson et al., 2001; Gibala et al., 2006). Despite these known physiological adaptations, the rate of oxygen extraction in muscle (represented by change in deoxyhaemoglobin: Δ[HHb]) or tissue saturation index (TSI), is not usually influenced by short training interventions (i.e., <6 weeks) (Overend et al., 1992; Berger et al., 2006; McKay et al., 2009); whether SIT can elicit adaptations at the muscular level following a shorter period of training is inconclusive.

The direct relationship between pulmonary and muscle oxygen extraction is important in understanding central (i.e., adaptations to cardiorespiratory function rather than adaptations to the peripheral vasculature and trained muscle) and peripheral (i.e., adaptations within skeletal muscle such as capillarization and/or mitochondrial biogenesis) adaptations to SIT; both parameters provide a measure of endurance capacity in recreationally active individuals (Murias et al., 2011; Spencer et al., 2013). Research in men suggests that an improvement in τV̇O_2_*_*p*_* with no concurrent change in TSI following a period of exercise training indicates that increased muscle oxygen utilization is accompanied by faster muscle oxygen extraction (MacPhee et al., 2005; McKay et al., 2009; Murias et al., 2011; Spencer et al., 2013). While research in women has found similar effects, hormone status or OC use has not been previously considered (Talanian et al., 2007; Astorino et al., 2011; Murias et al., 2011). Due to the potential influence of OC use on cardiovascular function and skeletal muscle blood flow due to chronic exogenous oestradiol and progestogen exposure, as well as our previous finding that OC use dampens V̇O_2__peak_ adaptations to SIT in women (Schaumberg et al., 2017a), the investigation of whether OC use influences pulmonary (τV̇O_2_*_*p*_*) and muscular oxygen extraction (TSI), as well as associated cardiovascular adaptation to SIT is warranted.

Therefore, the aim of the present study was to investigate the influence of OC use on pulmonary, cardiovascular and muscular oxygen uptake kinetics adaptation at moderate and heavy exercise intensities following 4 weeks of SIT.

## Materials and Methods

### Overview

Physically active women with either regular menstrual cycles (MC; no current hormonal contraception) or using an OC completed two exercise tests – an incremental exercise test and a square-wave step-transition protocol (separated by a minimum of 48 h), prior to, and following a 4-week SIT program.

### Participants

Healthy, recreationally active women (regularly completing at least 150 min of self-reported moderate to vigorous physical activity per week, but not currently training for, or competing at state or national level sport competition), who were either long-term (minimum 6 months uninterrupted) monophasic combined OC users (*n* = 25) or experiencing regular natural menstrual cycles (MC; *n* = 22) participated in the study. All experimental procedures were approved by an ethics committee of The University of Queensland and participants provided written informed consent.

### Control Measures

The procedures relating to hormone verification and analysis and body composition assessment are described in detail by Schaumberg et al. (2017b). To summarize, all OC users completed testing in the ‘active pill’ phase of the oral contraceptive cycle, and all naturally menstruating women completed testing in the mid-luteal phase of the menstrual cycle with serum hormone verification conducted at each timepoint. Nutrition, hydration, and exercise control measures have also been previously described (Schaumberg et al., 2017a). In brief, prior to each trial participants completed a 24-h food diary, fasted overnight, consumed a standardized moderate carbohydrate pretrial meal 1 h before arrival at the laboratory, abstained from caffeine, alcohol, and other stimulants and depressants for 24 h, recorded any additional medications or supplements, and maintain an euhydrated state. Participants were encouraged to maintain their normal physical activity levels throughout the study; however, were asked to refrain from strenuous physical activity for 24 h before each trial and arrive at the laboratory in a rested state. A pretrial preparation checklist was completed to confirm compliance to pretesting requirements.

### Experimental Protocol

**FIGURE 1:**
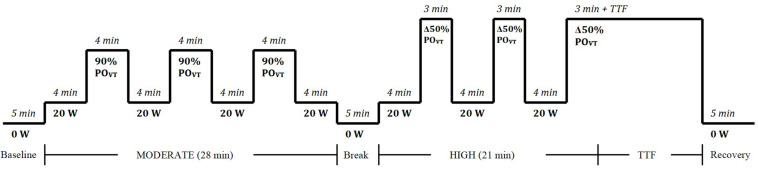
Square-wave step transition exercise protocol to moderate and heavy exercise intensities. W, watts; PO_VT_, power output at ventilatory threshold; min, minute; TTF, time to fatigue.

In each of the experimental trials, participants completed 3, 4-min step transitions to a moderate exercise intensity [90% of power output at ventilatory threshold (PO_VT_); calculated as 90% of the power output (PO) in Watts (W) achieved at ventilatory threshold (VT)] and three, 3-min transitions to a high exercise intensity [Δ50% PO_VT_; calculated as PO at VT plus 50% of the difference between PO at VT and peak power output (PPO)], as determined from the incremental exercise test (Joyce et al., 2013; Stanley et al., 2014) (Figure 1). The first transition was preceded by 4 min of baseline cycling (20 W) and each subsequent transition was separated by 4 min of baseline cycling (20 W). Participants were instructed to maintain a consistent cadence (70 ± 10 RPM) throughout the baseline, moderate, and high intensity cycling. Heart rate (HR) (Suunto^®^, United States), expired air (Parvo Medics’ TrueOne^®^ 2400 Indirect Calorimetry System, Utah, United States), cardiovascular parameters (PhysioFlow, Manatec Biomedical, France), and muscle oxygenation via near infra-red spectroscopy (NIRS; Portalite, Artinis Medical Systems BV, Netherlands) were continuously monitored throughout the trial, and RPE (Borg, 1973) was recorded at the end of every step-transition.

### Measures

#### Respiratory Measures

A familiarization session was completed prior to the first experimental trial. The V̇O_2__peak_ protocol involved continuous incremental (25 W⋅min^–1^) exercise test on an electronically braked cycle ergometer (Lode Excalibur Sport, Lode BV, Groningen, Netherlands) as previously described (Schaumberg et al., 2017a). Respiratory gas exchange was continuously recorded via automated indirect calorimetry (Parvo Medics’ TrueOne^®^ 2400 Indirect Calorimetry System, Utah, United States) for calculation of ventilatory parameters. Before each test, the analyzers were calibrated in accordance with the manufacturers’ recommendations. From the incremental test, data were averaged in 15-s epochs; V̇O_2__peak_ was defined as the highest V̇O_2_ value attained during a 15-s period (Rossiter et al., 2006; Stanley et al., 2014). During the experimental trials, average V̇O_2_ was determined from 5-s interval data.

#### Cardiovascular Measures

During exercise, heart rate (HR), stroke volume (SV) and cardiac output (Q̇) were measured continuously using impedance cardiology (PhysioFlow^®^, Manatec Biomedical, France) (Charloux et al., 2000; Richard et al., 2001). The theoretical basis for determining cardiac output from this method and its validity during rest and exercise has been described previously (Charloux et al., 2000; Lepretre et al., 2004), and has been validated against the direct Fick method (Richard et al., 2001). Two sets of electrodes (Skintact FS-50, Leonhard Lang Gmbh, Austria) – one transmitting, one sensing – were applied above the supra-clavicular fossa at the left base of the neck, and along the xiphoid process. Another two electrodes were used to monitor a single electrocardiographic signal (ECG; CM5 position). Blood pressure was assessed (Digital blood pressure monitor, UA-767, A&D Instruments Ltd., United Kingdom) as part of standard calibration process for the PhysioFlow^®^ prior to the exercise test. During the experimental trials (Figure 1), HR, SV, and Q̇ data were sampled at 5-s intervals, with the average for each interval determined during moderate and high loads. The coefficient of variation of cardiac output measures during peak exercise using this method has been reported as 3.4–3.6% (Hsu et al., 2006).

#### Near-Infrared Spectroscopy Measurements

Near-infrared spectroscopy (NIRS) (Portalite, Artinis Medical Systems BV, Netherlands) estimated the oxygenation of the right vastus lateralis muscle during the performance trial. A three-wavelength continuous wave system was used, which simultaneously used the modified Beer–Lambert and spatially resolved spectroscopy methods (Ihsan et al., 2013; Stanley et al., 2013). Changes in total hemoglobin (t_Hb_) oxyhaemoglobin (O_2_Hb) and deoxyhaemoglobin (HHb) were measured using the differences in absorption characteristics of light at 775, 810, and 850 mm (Stanley et al., 2013). An arbitrary value for the differential path length of 3.83 mm was used due to the uncertainty of proton path length at rest and during exercise (Stanley et al., 2013).

The NIRS device was connected via Bluetooth to a computer acquiring data at 10 Hz for later analysis. The probe was positioned one-third of the way along the vertical length of the thigh (from the quadriceps tendon on the patella). The NIRS device was wrapped in a zip-locked bag for water-proofing and covered in black material to prevent slipping and interference from ambient light and strapped to the leg securely with a bandage. Changes in t_Hb_, O_2_Hb, and HHb were reported as a change from baseline measures after first recording the tissue saturation index (TSI). The balance between oxygen consumption of the muscle tissue and oxygen supply (Equation 1) represented TSI (as a percentage).

(1) $$\text{TSI}=\left[{\text{O}}_{2}\,\mathrm{Hb}\,\mathrm{consumed}\right]/\left[{\text{O}}_{2}\,\mathrm{Hb}\,\mathrm{supplied}\right]\times \left[\text{HHb}\right]\times 100$$

Equation 1: TSI expressed as changes in O_2_Hb and HHb (Stanley et al., 2013). TSI, tissue saturation index; O_2_Hb, oxygenated hemoglobin; HHb, deoxygenated hemoglobin.

### Sprint Interval Training Protocol

As previously described (Schaumberg et al., 2017a), the SIT protocol required participants to complete three supervised SIT sessions per week for 4 weeks, with a minimum of 36 h between sessions. Each session involved 1 min of work followed by 2 min of passive recovery in a 1:2 work:rest ratio (Ready et al., 1981; Gosselin et al., 2012). The work interval intensity was self-selected at the maximal sustainable effort between 100 and 120% of PPO determined in the V̇O_2__peak_ test. Participants completed 10 1-min repetitions per session and peak heart rate, RPE, average power output and PPO were recorded. All exercise sessions were completed on an air- and magnetically braked cycle ergometer (Wattbike Ltd., Nottingham, United Kingdom).

### Data Analysis

#### Assessment of Pulmonary Oxygen Uptake Kinetics

Recorded data for $\dot{\text{V}}$O_2_ during each 4-min moderate (90% PO_VT_) and 3-min high (Δ50% PO_VT_) (plus an additional 1 min prior to each interval to determine baseline $\dot{\text{V}}$O_2_) loads, were interpolated into 5 s intervals; aberrant data points (caused by swallowing and coughing) were filtered out (Ozyener et al., 2001; Berger et al., 2006). To decrease the signal-to-noise ratio caused by high variability between breaths, the common practize of including multiple exercise transitions into the same protocol was employed (Whipp et al., 2005; Stanley et al., 2014). The first interval of each intensity was excluded from analysis due to the lack of a priming effect (Whipp et al., 2005; DeLorey et al., 2007; Stanley et al., 2013). Therefore, $\dot{\text{V}}$O_2_ recorded during the second and third intervals for each intensity of each experimental trial was time synchronized and ensemble averaged to yield a single response for each participant for each trial. A repeated iterative technique (Sigmaplot 10, SPSS Science; Chicago, IL, United States) and a mono-exponential function (Equation 2) (Ozyener et al., 2001; Dorado et al., 2004; Whipp et al., 2005; Stanley et al., 2014) were used to model τ$\dot{\text{V}}$O_2__p_ over moderate and high loads.

(2) $$\dot{\text{V}}\,{\text{O}}_{2\,p}\,\left(\text{t}\right)=\dot{\text{V}}\,{\text{O}}_{2}\,\text{baseline}+\text{Ampl}\times \left[1-{\text{e}}^{-\left(\tau -\text{TD}/\tau_{\text{on}}\right)}\right]\times {\text{U}}_{1}$$

Equation 2: Mono-exponential function (Sigmaplot 10, SPSS Science; Chicago, IL, United States) where *u* = if {*t* < TD1, VO2b, VO2b + A1^∗^[1−exp(−(t-TD1/*tau*1)]}. Initial parameters $\dot{\text{V}}$O_2__b_ = 0.5 TD1 (time delay) = −0.5; A1 (amplitude) = 1.5; Tau1 = 30. Constraints VO2 > 0; A1 > 0; Tau1 > 0; TD1 ≥ 0. $\dot{\text{V}}$O_2__p_, pulmonary oxygen kinetics; $\dot{\text{V}}$O_2_, oxygen consumption; Ampl, amplitude; TD, time delay.

In Equation 2 $\dot{\text{V}}$O_2_ baseline is the average $\dot{\text{V}}$O_2_ during the 60 s prior to onset of the rest- (or active recovery) to-exercise transition, Ampl is the asymptotic amplitude for the exponential term and τon is the time constant of the exponential (seconds) (Stanley et al., 2014). Use of the mono-exponential function was most appropriate due to its simplicity when considering the rare occurrence of τ$\dot{\text{V}}$O_2__p_ phase III (due to the priming effect from the first interval, the short duration of the interval and the submaximal intensity of exercise, therefore if Phase III were to occur the amplitude would be minor) (Stanley et al., 2014). Phase I to Phase II transition occurred approximately 15 s after the onset of exercise for all participants (determined by visual examination), therefore this initial cardiodynamic component was excluded by deleting the first 20 s of data (Ozyener et al., 2001; Dorado et al., 2004; Stanley et al., 2014). Overall τ$\dot{\text{V}}$O_2__p_, *tau*1 and mean response time (MRT = time delay + *tau*1) were calculated and very low residuals (*r*^2^ > 0.98) obtained (Stanley et al., 2014), providing an overall description of on-transient oxygen uptake kinetics. While *tau*1 and MRT are closely related, MRT has been shown to be more reliable that *tau*1 in recreationally active women (Schaumberg et al., unpublished data), and therefore MRT was used as the primary measure of interest.

#### NIRS Data and Assessment of De-Oxygenation Rates

Data from the NIRS device (t_Hb_, O_2_Hb, and [HHb]) were sampled down from 10 to 1 Hz and then averaged into 5 s intervals. Since tissue saturation index has been shown to provide a more accurate indication of muscle oxygenation status than Δ[HHb] (Wolf et al., 2007), TSI data was modeled. Using the Sigmaplot 10 program (SPSS Science; Chicago, IL, United States), a linear model was used to calculate the results from the average of the second and third intervals for both moderate and high intensity. Similar to τ$\dot{\text{V}}$O_2__p_, the first interval was excluded from analysis due to the priming effect (Stanley et al., 2013). Moderate exercise prior to heavy workloads has been shown to influence Δ [HHb] (Whipp et al., 2005; Spencer et al., 2013), therefore, both workloads were analyzed using a linear model (Equation 3) (Bae et al., 2000):

(3) TSI=a×t+b

Equation 3: Linear equation (Sigmaplot 10, SPSS Science; Chicago, IL, United States). TSI, tissue saturation index; *t*, time; *a*, slope; *b*, *y*-intercept.

TSI was modeled without time delay during the first 20 s of moderate intensity and 30 s of high intensity. However, the slope (*a*) was retained as an index of deoxygenation rate (Stanley et al., 2013). This particular model yielded very low residuals (*r*^2^ > 0.98) and provided an overall descriptor of the muscular deoxygenation rate, and therefore muscle deoxygenation kinetic response to exercise.

#### Assessment of Cardiovascular Kinetics

Heart rate and Q̇ on-transient kinetics were modeled using the same iterative technique adopted for $\dot{\text{V}}$O_2_ on-transient kinetics. HR and Q̇ data were fitted with a mono-exponential function consistent with Equation 2 (using the same 4 or 5 min window), with the HR and Q̇ data substituted for $\dot{\text{V}}$O_2_ (Stanley et al., 2014). Unlike the $\dot{\text{V}}$O_2_ on-transient kinetics analysis, the initial 20 s of data was not deleted due to lack of a cardiodynamic (Phase I to Phase II) transition. The mean response time (MRT = time delay + *tau*1) was calculated to provide an overall description of on-transient cardiovascular kinetics.

### Statistical Analysis

Data were analyzed using Microsoft Excel^®^ 2007 and SPSS^®^ (version 22.0, SPSS, Inc., Chicago, IL, United States). Normality of distribution was tested using the Kolmogorov–Smirnov test; when not normally distributed, data were log-transformed and re-checked for normality of distribution. Analyses included standard descriptive statistics, paired *t*-test, and two-way repeated measures analysis of variance (ANOVA) (with a main effect for training × group). To locate the source of significant differences, the Bonferroni *post hoc* test was used. Homogeneity of variance was confirmed using Mauchly’s test of sphericity. When the assumption of sphericity was violated (*p* < 0.05), the *F*-statistic was adjusted using the Greenhouse–Geisser correction. Where Mauchly’s test of sphericity was not found to be significant, *post hoc* analyses assumed sphericity (Vincent, 1999). Magnitude-based inferences (Hopkins et al., 2009; Batterham and Hopkins, 2015) calculated the between-trial standardized differences or effect sizes [ES, 95% confidence interval (CI)] using the pooled standard deviation (Cohen, 1988) and standard threshold values (Batterham and Hopkins, 2005). All tests were two-tailed and statistical significance was set at *p* < 0.05. Parametric results are given as the mean, standard deviation and 95% confidence interval (CI), [mean ± SD (95% CI)]; non-parametric results are given as the median and interquartile range and 95% CI, [median (IQR) (95% CI)] unless stated otherwise.

## Results

### Participant Characteristics, Control Measures, and Training Protocol

Participant recruitment and retention has previously been described (Schaumberg et al., 2017a). Due to the nature of the outcome measures, we included all naturally menstruating participants within the main data set, including six of the 22 MC participants who had ovulatory, regular menstrual cycles but exhibited potential luteal phase deficiency (LPD) based on failing to meet the serum progesterone criterion of >6 ng.mL^–1^ (Schaumberg et al., 2017b). Supplementary Table S1 comparing the normal MC (*n* = 16) versus the potential LPD MC (*n* = 8) participants has been included; no significant differences between outcome measures pre- or post-training were found, though the MC LPD group showed dampened pulmonary oxygen up-take kinetic responses to training, discussed below. All 25 participants recruited to the OC group were taking a low-dose, monophasic combined oestradiol and progestin formulation. There were variations in androgenic (*n* = 5), antiandrogenic (*n* = 5) and non-androgenic (*n* = 15) formulations [calculated using the method of Greer et al. (2005)]; subsequent analyses confirmed androgenicity of OC type did not influence baseline characteristics or outcome measures. Physical activity, energy intake and body composition parameters were not different within or between groups at any timepoint, however, due to the inclusion of the potential LPD participants (*n* = 8) there were some differences in hormone concentrations between groups that have been previously described (Schaumberg et al., 2017b), with the MC group having higher oestradiol, progesterone and free androgen index (*p* < 0.001), and lower sex-hormone binding globulin (SHBG; *p* < 0.001). As such, participant characteristics are re-presented in Table 1.

**TABLE 1:** Participant characteristics.

	Oral contraceptive group (*n* = 25)		Menstrual cycle group (*n* = 22)	
	Pre-training	Post-training	Pre-training	Post-training
**Participant demographics and control measures**				
Age (years)	25.5 ± 5.4 (23.1–27.8)	X	26.4 ± 5.2 (24.0–28.8)	X
MC length (days)	28.0 ± 0.0 (28.0–28.0)	X	30.5[28.8–33.3]* (29.9–32.6)	X
Physical activity (min.wk^–1^)	247 ± 64 (222–272)	235 ± 61 (211–258)	235 ± 58 (211–260)	222 ± 51 (201–243)
Energy intake (kJ.kg^–1^.d^–1^)	8461 ± 3194 (6896–10026)	8490 ± 2452 (7103–9877)	8373 ± 2360 (7179–9567)	8499 ± 1971 (7269–9712)
**Body composition**				
Body mass (kg)	63.6 ± 7.8 (60.3–66.8)	63.4 ± 7.2 (60.4–66.3)	64.6 ± 9.2 (60.5–68.8)	64.2 ± 8.8 (60.2–68.2)
Body mass index (kg.m^–2^)	22.6 ± 2.1 (21.7–23.4)	22.6 ± 2.1 (21.7–23.4)	22.7 ± 2.3 (21.7–23.7)	22.7 ± 2.3 (21.7–23.7)
Fat mass (kg)	20.7 ± 4.8 (18.7–22.7)	20.5 ± 4.6 (18.6–22.4)	21.0 ± 5.2 (18.8–23.3)	20.8 ± 5.6 (18.2–23.3)
Lean body mass (kg)	40.6 ± 4.4 (38.8–42.5)	40.6 ± 4.1 (38.9–42.3)	41.3 ± 5.9 (38.7–43.9)	41.2 ± 5.2 (38.8–43.5)
Lean body mass – legs (kg)	13.6 ± 1.8 (12.9–14.4)	13.8 ± 1.9 (13.0–14.5)	13.8 ± 2.5 (12.7–14.9)	13.8 ± 2.3 (12.7–14.8)
Body fat (%)	32.3 ± 4.8 (30.3–34.3)	32.1 ± 4.8 (30.1–34.0)	32.3 ± 5.3 (30.0–34.7)	32.0 ± 5.9 (29.3–34.7)
**Hormone measures**				
Estradiol (pg.mL^–1^)	5.6 [5.0–10.3] (5.7–13.5)	5.1[5.0–10.2] (11.0–63.8)	124.4 ± 67.5^Λ^ (92.8–156.1)	93.2 ± 67.5^#Λ^ (62.4–123.9)
Progesterone (ng.mL^–1^)	0.6 ± 0.3 (0.4–0.7)	0.5 ± 0.3 (0.4–0.6)	10.0 ± 7.9^Λ^ (6.3–13.7)	1.0[0.7–1.4]^#^ (0.2–7.0)
Total testosterone (ng.mL^–1^)	0.20 ± 0.10 (0.10–0.20)	0.13 ± 0.07 (0.10–0.16)	0.2[0.1–0.4] (0.2–0.3)	0.3 ± 0.2^Λ^ (0.2–0.4)
SHBG (pg.mL^–1^)	209.0 ± 87.1 (170.3–247.6)	189.6 ± 99.3 (145.6–233.7)	62.6[40.8–90.0]^Λ^ (50.7–86.3)	58.5[35.6–81.9]^Λ^ (47.5–76.9)
Free androgen index (%)	7.8[4.2–8.5] (6.9–14.7)	8.6 ± 5.6 (6.1–11.0)	33.2[14.7–77.7]^Λ^ (22.3–99.4)	55.3[19.6–96.5]^Λ^ (35.3–106.0)

Parametric data are presented as mean ± SD (95%CI); non-parametric data are presented as median [IQR] (95% CI). ^#^p < 0.05 vs. pre-training; *p < 0.001 vs. pre-training; ^Λ^p < 0.05 vs. OC group.

### Peak Exercise Responses and Time to Fatigue

**FIGURE 2:**
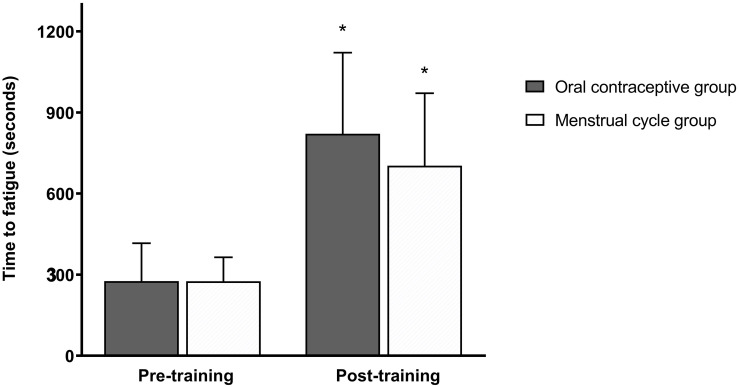
Time to fatigue (seconds) at a heavy intensity (Δ50%PO_VT_) for the oral contraceptive group (*n* = 25) and menstrual cycle group (*n* = 22) pre- and post-training. Data are mean ± SD. *p < 0.001 vs. pre-training; paired samples *t*-test.

Peak exercise adaptations have previously been reported (Schaumberg et al., 2017a), with the OC group showing dampened peak exercise adaptations to SIT compared to the MC group. There was no significant difference in time-to-fatigue (TTF) between groups at any timepoint (*p* > 0.05) (Figure 2). Following training, TTF was increased from pre-training in both groups (*p* < 0.001). Standardized between-group differences for within-group changes (Cohen’s *d*) demonstrated that there was a likely higher TTF adaptation to training in the OC-group compared to the MC-group (0.96 ± 1.04).

### Pulmonary Oxygen Uptake Kinetics

**FIGURE 3:**
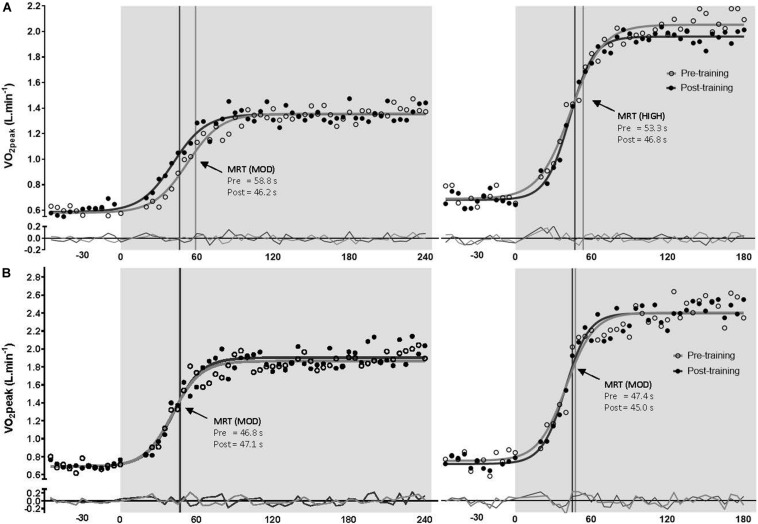
Pulmonary oxygen uptake profile (with model best fit line and residuals) for a representative naturally menstruating participant **(A)** and oral contraceptive using participant **(B)** during the transition to moderate (left) and heavy (right) intensity exercise, pre-training (open circles and gray line), post-training (closed circles and black line). Mean response time (MRT) is indicated by a matched vertical line. Shading represents exercise onset.

There was no significant difference between groups for τ$\dot{\text{V}}$O_2__p_ [expressed as the mean response time (MRT) in seconds] at any time point at moderate or high intensity (*p* > 0.05). Following training, τ$\dot{\text{V}}$O_2__p_ was improved in the MC-group at both moderate (*p* = 0.021) and high (*p* = 0.015) intensities but the OC-group showed no change from baseline at both intensities (*p* > 0.05). When the MC-group was sub-grouped for potential LPD, the LPD group showed dampened τ$\dot{\text{V}}$O_2__p_ at both intensities (*p* < 0.05) as measured by MRT; but there was no significant group × time interaction between normal MC and LPD MC (see Supplementary Table S1). There was a significant group x time interaction for τ$\dot{\text{V}}$O_2__p_ at moderate intensity (*p* = 0.020); the interaction was approaching significance at high intensity (*p* = 0.086). *Post hoc* analyses suggested that the MC-group showed greater improvement in τ$\dot{\text{V}}$O_2__p_ at high intensity following training compared to the OC-group (OC-group −1.4 s vs. MC-group −6.1 s; *p* = 0.021); but this was not significant between groups at moderate intensity (OC-group +0.5 s vs. MC-group −4.4 s; *p* = 0.097). Data are presented in Table 2 and the τ$\dot{\text{V}}$O_2__p_ profile at both intensities from a representative participant is presented in Figure 3. Standardized between-group differences for within-group changes (Cohen’s *d*) demonstrated that the OC-group had a likely lower τ$\dot{\text{V}}$O_2__p_ adaptation to training compared to the MC-group at moderate (−0.54 ± 0.67) and high (−0.57 ± 0.49) intensities.

**TABLE 2:** Pulmonary oxygen uptake, cardiac output, and heart rate on-kinetic responses to moderate and heavy intensity exercise, pre- and post-training in oral contraceptive users (*n* = 25) and naturally menstruating women (*n* = 22).

	Oral contraceptive group (*n* = 25)		Menstrual cycle group (*n* = 22)	
	Pre-training	Post-training	Pre-training	Post-training

Moderate intensity exercise (90% PO_VT_)				
**Pulmonary oxygen uptake on-kinetic response**				
Baseline $\dot{\text{V}}$O_2_ (L.min^–1^)	0.8 ± 0.1(0.7–0.8)	0.7 ± 0.1(0.6–0.7)^#^	0.7 ± 0.1(0.7–0.8)	0.7 ± 0.1(0.6–0.7)
Amplitude (L.min^–1^)	0.8 ± 0.2(0.7–0.9)	0.8 ± 0.2(0.7–0.9)	1.0 ± 0.5(0.7–1.2)	0.7 ± 0.4(0.6–0.9)^#^
Time delay (sec)	23.2 ± 7.3(20.1–26.3)	27.1 ± 8.9(23.4–30.9)	22.0 ± 8.0(18.3–25.6)	26.0 ± 9.3(21.8–30.2)
Time constant (*tau*1; sec)	32.2 ± 10.5(27.7–36.6)	26.9 ± 9.1(23.0–30.7)	36.0 ± 12.0(30.5–41.4)	25.9 ± 9.6(21.5–30.3)^#^
Mean response time (sec)	55.4 ± 6.7(52.5–58.2)	54.0 ± 7.8(50.7–57.3)	58.0 ± 10.1(53.4–62.6)	51.9 ± 5.5(49.3–54.4)^#^
**Cardiac output on-kinetic response**				
Baseline Q̇ (L.min^–1^)	11.0 ± 1.3(10.5–11.6)	12.3 ± 2.2(11.3–13.3)*	11.0 ± 1.6(10.2–11.7)	10.6 ± 1.0(10.2–11.1)
Amplitude (L.min^–1^)	6.6 ± 3.1(5.2–8.0)	5.2 ± 1.2(4.7–5.8)^#^	4.0 ± 1.3(3.4–4.6)	4.6 ± 1.5(3.9–5.3)^#^
Time delay (sec)	7.2 ± 4.0(5.4–9.0)	4.0 ± 1.9(3.1–4.8)	6.0 ± 2.5(4.8–7.2)	8.8 ± 6.6(5.7–11.9)^#^
Time constant (*tau*1; sec)	45.9 ± 10.1(41.5–50.4)	38.4 ± 10.2(33.9–42.9)*	50.0 ± 10.7(45.0–55.0)	34.4 ± 10.5(29.5–39.3)*
Mean response time (sec)	53.1 ± 10.3(48.6–57.7)	38.1 ± 10.2(33.9–42.9)*	56.0 ± 10.0(51.3–60.7)	43.2 ± 5.8(40.5–45.9)*
**Heart rate on-kinetic response**				
Baseline heart rate (bpm)	105.5 ± 10.4(100.9–110.1)	106.8 ± 12.1(101.4–112.2)	113.8 ± 8.5(109.8–117.8)	107.3 ± 10.0(102.6–112.0)*
Amplitude (bpm)	44.6 ± 8.1(41.0–48.3)	39.9 ± 9.4(35.8–44.1)*	32.0 ± 6.8(28.8–35.2)	32.4 ± 9.6(27.9–36.9)
Time delay (sec)	4.1 ± 4.3(2.2–6.0)	2.8 ± 1.0(2.3–3.2)	4.2 ± 3.4(2.7–5.8)	6.9 ± 6.3(4.0–9.9)
Time constant (*tau*1; sec)	55.4 ± 9.1(51.4–59.5)	43.2 ± 9.9(38.8–47.6)*	53.1 ± 15.7(45.8–60.5)	38.2 ± 10.4(33.4–43.1)*
Mean response time (sec)	59.5 ± 10.4(54.9–64.1)	46.0 ± 10.2(41.5–50.5)*	57.3 ± 14.0(50.8–63.9)	45.1 ± 6.1(42.3–48.0)*

**Heavy intensity exercise (Δ50% PO_VT_)**				

**Pulmonary oxygen uptake on-kinetic response**				
Baseline $\dot{\text{V}}$O_2_ (L.min^–1^)	0.9 ± 0.2(0.8–0.9)	0.8 ± 0.1(0.7–0.8)	0.8 ± 0.2(0.8–0.9)	0.8 ± 0.1(0.8–0.9)
Amplitude (L.min^–1^)	1.3 ± 0.3(1.2–1.4)	1.3 ± 0.3(1.2–1.4)	1.3 ± 0.4(1.1–1.5)	1.3 ± 0.4(1.1–1.4)
Time delay (sec)	18.2 ± 7.2(15.2–21.3)	22.7 ± 5.9(20.2–25.3)^#^	20.6 ± 5.9(17.9–23.3)	23.1 ± 5.4(20.7–25.6)
Time constant (*tau*1; sec)	32.5 ± 10.0(28.3–36.7)	28.5 ± 10.0(24.3–32.7)	33.5 ± 8.5(29.7–37.4)	26.6 ± 9.0(22.5–30.7)^#^
Mean response time (sec)	50.7 ± 7.6(47.5–53.9)	51.2 ± 8.0(47.9–54.6)	54.1 ± 8.9(50.1–58.2)	49.7 ± 6.9(46.6–52.9)^#^
**Cardiac output on-kinetic response**				
Baseline Q̇ (L.min^–1^)	13.0 ± 1.7(12.3–13.8)	14.2 ± 1.7(13.5–15.0)^#^	12.7 ± 1.0(12.3–13.1)	12.2 ± 1.0(11.8–12.7)^#^
Amplitude (L.min^–1^)	6.7 ± 2.0(5.8–7.6)	6.7 ± 2.1(5.7–7.6)	5.4 ± 1.6(4.7–6.2)	6.1 ± 1.5(5.4–6.8)^#^
Time delay (sec)	5.8 ± 4.4(3.8–7.7)	9.7 ± 3.8(8.0–11.4)^#^	8.7 ± 3.7(7.0–10.4)	9.9 ± 5.0(7.6–12.2)
Time constant (*tau*1; sec)	45.4 ± 9.4(41.3–49.6)	31.8 ± 6.6(28.9–34.8)*	44.3 ± 13.8(37.9–50.8)	30.0 ± 10.1(25.2–34.7)*
Mean response time (sec)	51.2 ± 9.9(46.8–56.0)	41.5 ± 6.8(38.5–44.5)*	53.0 ± 13.8(46.6–59.5)	39.9 ± 6.3(36.9–42.8)*
**Heart rate**				
Baseline heart rate (bpm)	122.8 ± 7.8(119.4–126.3)	120.3 ± 10.1(115.8–124.8)	125.9 ± 10.5(121.0–130.8)	120.7 ± 11.0(115.6–125.8)^#^
Amplitude (bpm)	56.2 ± 8.2(52.6–59.9)	52.9 ± 8.0(49.4–56.5)*	49.0 ± 10.9(44.0–54.1)	48.1 ± 10.5(43.2–53.0)
Time delay (sec)	7.4 ± 5.3(5.0–9.7)	5.1 ± 3.8(3.4–6.8)^#^	10.3 ± 3.1(8.8–11.7)	6.8 ± 1.9(5.9–7.7)*
Time constant (*tau*1; sec)	45.2 ± 6.1(42.5–47.9)	43.6 ± 10.9(38.8–48.4)	43.4 ± 9.8(38.8–48.0)	41.3 ± 4.9(39.0–43.6)
Mean response time (sec)	52.6 ± 10.1(48.1–57.0)	48.7 ± 12.2(43.3–54.1)*	53.7 ± 9.9(49.1–58.3)	48.1 ± 5.0(45.8–50.4)*

PO_*VT*_, power output at ventilatory threshold; sec, seconds; $\dot{\text{V}}$O_2_, volume of oxygen consumed. Q̇, cardiac output; sec, seconds. Parametric data are presented as mean ± SD (95%CI); non-parametric data are presented as median [IQR] (95% CI). ^#^p < 0.05 vs. pre-training; *p < 0.001 vs. pre-training.

### Cardiac Output Kinetic Response

There were no differences between groups for cardiac output kinetic response represented as mean response time (Q̇_MRT_) pre- or post-training at either intensity (*p* > 0.05). Following training, Q̇_MRT_ improved in both groups at both intensities (*p* < 0.001). There was a significant group x time interaction for Q̇_MRT_ at moderate (*p* = 0.003) and high (*p* = 0.039) intensity. *Post hoc* analyses suggested there was no difference between groups for pre- to post-training improvements in Q̇_MRT_ at moderate [OC-group −15.0 s vs. MC-group −12.8 s; *p* = 0.405], and high intensities [OC-group −9.7 s vs. MC-group −13.1 s; *p* = 0.282]. Data are presented in Table 2 and the cardiac output profile at both intensities from a representative participant is presented in Supplementary Figure S1. Standardized between-group differences for within-group changes (Cohen’s *d*) demonstrated that there was no clear between-group difference in Q̇_MRT_ adaptation to training at both moderate (0.19 ± 0.48) and heavy (0.29 ± 0.53) exercise intensities (Table 3).

**TABLE 3:** Standardized between-group differences for within-group changes for the oral contraceptive group versus the menstrual cycle group following training and de-training.

	Cohen’s *d* ± SD	95% CI	% chance for OC change to be higher/trivial/lower than MC	Descriptive of difference
Time to fatigue (sec)	0.96 ± 1.04	−0.08–2.01	93/6/1	Likely higher
**Moderate intensity (90% PO_VT_)**				
Mean response time (sec)	0.54 ± 0.67	−0.13–1.21	85/14/2	Likely higher
Tissue saturation index (slope)	−1.03 ± 0.71	−1.74–−0.33	0/1/99	V. likely lower
Mean response time (Q̇)	0.19 ± 0.48	−0.29–0.68	49/46/5	Unclear
Mean response time (HR)	−0.10 ± 0.46	−0.56–0.36	10/57/33	Unclear
**Heavy intensity (Δ50% PO_VT_)**				
Mean response time (sec)	0.57 ± 0.49	0.08–1.07	93/6/0	V. likely higher
Tissue saturation index (slope)	−0.66 ± 0.41	−1.07–−0.25	0/1/99	V. likely lower
Mean response time (Q̇)	0.29 ± 0.53	−0.25–0.82	63/34/4	Unclear
Mean response time (HR)	0.17 ± 0.50	−0.32–0.67	45/48/7	Unclear

*SD, standard deviation; CI, confidence interval; OC, oral contraceptive group; MC, naturally menstruating group; sec, seconds; Q, cardiac output; HR, heart rate; PO_*VT*_, power output at ventilatory threshold; V. likely, very likely.*

### Heart Rate Kinetic Response

There was no difference between groups for HR_MRT_ at any time point at either intensity (*p* > 0.05). Following training, HR_MRT_ improved in both groups and at each intensity (*p* < 0.05). There was no significant group × time interaction for HR_MRT_ at either moderate (*p* = 0.269) or high intensity (*p* = 0.712). Data are presented in Table 2 and the heart rate profile at both intensities from a representative participant is presented in Supplementary Figure S2. Standardized between-group differences for within-group changes (Cohen’s *d*) demonstrated no clear differences between groups (Table 3).

### Muscle Deoxygenation Rates

**FIGURE 4:**
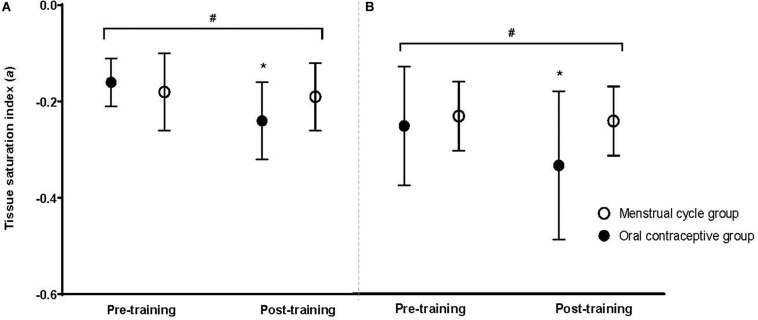
Tissue saturation index at a moderate exercise intensity **(A)** and a high exercise intensity **(B)** for the oral contraceptive group (*n* = 25) and menstrual cycle group (*n* = 22) pre- and post-training. Data are mean ± SD. *p < 0.001 vs. pre- training; ^#^*p* < 0.05 group × time interaction.

There was no difference between groups for TSI (presented here as an index/percentage) at baseline for either intensity (moderate: MC −0.18 ± 0.08; OC −0.16 ± 0.05; high: MC −0.24 ± 0.07; OC −0.26 ± 0.12; all *p* > 0.05). Post-training, the OC-group had improved TSI compared to the MC-group at both moderate (OC −0.24 ± 0.08; MC −0.19 ± 0.07; *p* = 0.027) and high (OC −0.34 ± 0.14; MC −0.25 ± 0.07; *p* = 0.018) intensities. Following training, TSI was improved in the OC-group at both intensities [moderate: Δ−0.08 (50%); high: Δ−0.08 (30.8%); both *p* < 0.001]; but did not change in the MC-group at either intensity [moderate: Δ−0.01 (6.0%), *p* = 0.295; high: Δ−0.01 (4.1%) *p* = 0.422]. There was a significant group × time interaction for TSI at moderate (*p* = 0.001), and high (*p* < 0.001) intensities. *Post hoc* analyses suggested the OC-group showed greater improvement in TSI at both moderate (*p* = 0.004) and high (*p* = 0.002) intensities following training compared to the MC-group. Data are presented in Figure 4. Standardized between-group differences for within-group changes (Cohen’s *d*) demonstrated that the OC-group had a likely greater TSI adaptation to training compared to the MC-group at moderate (−1.03 ± 0.71) and heavy (−0.66 ± 0.41) intensities (Table 3).

## Discussion

The present study investigated the influence of OC use on time to fatigue and changes in pulmonary, cardiovascular and muscle oxygen uptake kinetics to sprint interval training (SIT). Pulmonary oxygen uptake kinetics (τ$\dot{\text{V}}$O_2__p_) as determined by MRT, improved following training in the MC-group only. However, tissue saturation index (TSI) improved in the OC-group only. Despite these differences, improvements in time to fatigue in response to SIT did not differ between groups.

Significant improvements (30%) in the mean response time for τ$\dot{\text{V}}$O_2__p_ at both moderate and high intensity exercise were observed with training in the MC-group only; the OC-group showed no significant change from baseline. When the MC-group was sub-grouped for potential LPD, the MC LPD sub-group also demonstrated dampened τ$\dot{\text{V}}$O_2__p_. The improvement in τ$\dot{\text{V}}$O_2__p_ observed in the MC-group is consistent with previous research, where Murias et al. (2011) demonstrated that τ$\dot{\text{V}}$O_2__p_ was improved in response to endurance training in young women. However, these authors did not include women using an OC and our results suggest that exogenous hormones may dampen the τ$\dot{\text{V}}$O_2__p_ adaptations to training. It is also possible that the absence of endogenous ovarian hormones (within the probable LPD group) may also dampen τ$\dot{\text{V}}$O_2__p_ adaptations. This is consistent with findings from our previous study (Schaumberg et al., 2017a), where $\dot{\text{V}}$O_2__peak_ improvement was lower in OC users compared to naturally menstruating women following training.

Improvement in τ$\dot{\text{V}}$O_2__p_ can increase exercise capacity at both moderate and higher intensities of exercise (Poole and Richardson, 1997). Oxygen delivery to skeletal muscle is challenged during high intensity exercise training, thereby eliciting structural adaptations. This leads to an increased blood flow and an increase in pulmonary and/or muscle oxygen kinetic responses at the onset of constant-load exercise (Poole and Richardson, 1997; Jones and Poole, 2005). Therefore, improvements in τ$\dot{\text{V}}$O_2__p_ may be limited by either central (e.g., cardiac output) or peripheral (e.g., skeletal muscle blood flow) adaptations (Barstow et al., 1990; Grassi, 2006). OC use has been shown to decrease collagen synthesis, and therefore capillarization, in response to exercise (Hansen et al., 2011). Therefore, reduced capillarization adaptation with exercise may dampen further central adaptations such as τ$\dot{\text{V}}$O_2__p_. In addition, OC use increases the release of hormones (e.g., growth hormone and other glucoregulatory hormones) (62), which can influence carbohydrate metabolism during exercise (Davidson and Holzman, 1973; Bunt, 1990; Bonen et al., 1991). As τ$\dot{\text{V}}$O_2__p_ is controlled intracellularly via oxidative phosphorylation, glucose is required to fuel the turnover of ATP for energy production (McKay et al., 2009). If there is limited blood glucose available, this could limit the improvement of τ$\dot{\text{V}}$O_2__p_. Luteal phase deficiency is a menstrual disturbance that may reflect early stages of low energy availability in women (De Souza et al., 2003). Therefore, the dampened τ$\dot{\text{V}}$O_2__p_ adaptation seen within the probable LPD group further support this potential mechanism.

Cardiac output on-kinetics improved in both groups at both moderate and high intensity loads following training. It is possible that this measure was not sensitive enough to detect any between-group differences. However, it is important to note that the adaptation of the women in this study was comparable to men, where it has previously been demonstrated that the heart rate time constant to moderate intensity exercise improves with just eight sessions of HIIT, and that end heart rate is also improved (McKay et al., 2009). Recent work by Howden et al. (2015) demonstrated that women had a markedly blunted cardiovascular response to 1 year of endurance training, compared to males. Therefore, further investigation into cardiovascular on-kinetic responses is warranted to explain the lack of adaptation in the present study.

The present study investigated TSI as an overall index of the muscle deoxygenation kinetics, representative of the dynamic balance between oxygen consumption of the muscle tissue and supply. While most literature reports total oxy- or deoxy-hemoglobin, TSI arguably provides a more effective measure of the overall muscular response (because of its incorporation of both oxy- and deoxy-hemoglobin and consumption and supply of oxygen), and is closely related to [HHB] (Ihsan et al., 2013).

The first, and most obvious, conclusion for the significant TSI improvement in the OC-group and no change in the MC-group is that the OC-group showed significant peripheral adaptation with training compared to the MC-group, yet, when analyzed in conjunction with the τ$\dot{\text{V}}$O_2__p_ adaptations, this assumption must be considered with caution, as TSI is a rate relative to both supply and utilization of oxygen at the muscle. TSI and τ$\dot{\text{V}}$O_2__p_ are often analyzed and reported together (Gurd et al., 2007; McKay et al., 2009; Murias et al., 2011), as TSI is indicative of peripheral adaptation whilst τ$\dot{\text{V}}$O_2__p_ is indicative of central adaptations (Murias et al., 2011). An increase in TSI (also demonstrated with the increase relative to exercise intensity) may indicate a mismatch between oxygen delivery and utilization during exercise (Spencer et al., 2013). As TSI adjusts more rapidly in response to exercise compared to τ$\dot{\text{V}}$O_2__p_ there is insufficient oxygen delivery for the working muscles and temporal dissociation occurs, which may result in greater reliance on anaerobic energy production at the working muscles (MacPhee et al., 2005; Spencer et al., 2013), and be potentially detrimental to exercise capacity.

The concomitant changes in TSI and τ$\dot{\text{V}}$O_2__p_ in the MC- and OC-groups can be further considered through the concept of a physiological phenomenon known as the ‘transient overshoot’ (Murias et al., 2011), whereby training instigates more efficient oxygen utilization as well as blood distribution, thereby causing a speeding of τ$\dot{\text{V}}$O_2__p_ with no change in TSI. An increase in muscle oxygen extraction causes insufficient oxygen delivery, instigating a temporal dissociation period between [HHB] and τ$\dot{\text{V}}$O_2__p_ (MacPhee et al., 2005; Spencer et al., 2013). This overshoot may be present with OC use, with greater utilization of oxygen at the muscle occurring after training due to peripheral adaptations, with concurrent blunting of oxygen supply indicated by τ$\dot{\text{V}}$O_2__p_.

Dynamic changes in NIRS-derived muscle deoxygenation (represented by TSI) provide insights into the balance between local muscle oxygen availability and utilization during exercise (Wolf et al., 2007; Spencer et al., 2013). During exercise muscle deoxygenation adjusts to an increased workload more rapidly than τ$\dot{\text{V}}$O_2__p_, resulting in a transient period characterized by an increased relative reliance on oxygen extraction to support a given metabolic rate. This temporary dissociation between adjustments of [HHB] and τ$\dot{\text{V}}$O_2__p_ suggest transient oxygen delivery insufficiency (Murias et al., 2013; Spencer et al., 2013) for the rate of oxygen consumption.

Therefore, we observed an abnormal response of τ$\dot{\text{V}}$O_2__p_ and TSI to exercise training in OC users, demonstrated by an apparent acceleration of TSI, which may suggest a mismatch of oxygen delivery and utilization at the exercising muscles. In our participant sample, oxygen delivery did not improve with training in OC users compared to naturally menstruating women, despite the apparent increase in local muscular oxygen utilization. Therefore, the next consideration based on these results is that exogenous hormones do not appear to influence peripheral adaptation to training and may indeed be beneficial. Indeed, the mechanism for the change in TSI in OC users warrants further investigation. Previous research has speculated the mismatch between oxygen utilization and delivery with OC use is due to its direct effect on the female sex hormones (estrogen and progesterone, and their exogenous forms). However, several other hormones (e.g., growth hormone, inflammatory factors, and free androgens) are also influenced by OC use and should therefore be considered. For example, growth factors, including growth hormone and free androgens are significantly different from naturally menstruating women; therefore it is necessary to further investigate this possible factor, due to their influence on capillarization and subsequent oxygen utilization at the muscle (Hansen et al., 2011; Hansen and Kjaer, 2014). Further, the influence of OC use on mitochondrial/oxidative enzymes is unknown, and further investigation into the potential influence of OC use on mitochondrial oxidative capacity is warranted.

## Conclusion

Exogenous ovarian hormones found in the oral contraceptive pill may be, at least in part, responsible for the dampened physiological adaptations to training in OC users. Although both OC users and naturally menstruating women improved TTF, we observed a dampened response of central physiological adaptation, demonstrated by pulmonary oxygen uptake kinetics in the OC group. This may have been offset by the greater improvements in muscle oxygen utilization in OC users, compared to the MC group. These results provide insight into potential mechanisms related to training adaptation in women. Based on these results, potential mechanisms may include the lack of endogenous ovarian hormones, as well as the influence of exogenous hormones on the overall endocrinological profile, including growth hormone and free androgens, may be implicated in dampening the physiological adaptations to training with OC use. These potential mechanisms warrant investigation to further elucidate the influence of OC use on adaptations to training in women.

## Data Availability Statement

The datasets generated for this study are available on request to the corresponding author.

## Ethics Statement

This study involving human participants was reviewed and approved by The University of Queensland Human Research Ethics Committee. The patients/participants provided their written informed consent to participate in this study.

## Author Contributions

MS, JS, DJ, XJ, LE, and TS designed the protocol and methods. MS and EH collected the data. MS, JS, and EH analyzed the data. All authors contributed to the writing and drafting of the manuscript and read and approved the manuscript.

## Conflict of Interest

The authors declare that the research was conducted in the absence of any commercial or financial relationships that could be construed as a potential conflict of interest.

## Supplementary Material

FIGURE S1 Cardiac output profile (with model best fit line and residuals; time in seconds on the *x*-axis) for a representative naturally menstruating participant **(A)** and oral contraceptive using participant **(B)** during the transition to moderate (left) and heavy (right) intensity exercise, pre-training (open circles and gray line) and post-training (closed circles and black line). Mean response time (MRT) is indicated by a matched vertical line. Shading represents exercise onset.

FIGURE S2 Heart rate profile (with model best fit line and residuals) for a representative naturally menstruating participant **(A)** and oral contraceptive using participant **(B)** during the transition to moderate (left) and heavy (right) intensity exercise, pre-training (open circles and gray line) and post-training (closed circles and black line). Mean response time (MRT) is indicated by a matched vertical line. Shading represents exercise onset.

TABLE S1 Pulmonary oxygen uptake, cardiac output and heart rate on-kinetic responses to moderate and heavy intensity exercise, pre- and post-training in naturally-menstruating women, sub-grouped for normal luteal phase characteristics (*n* = 16) and apparent luteal phase deficient women (*n* = 8); as determined by serum progesterone concentrations.
