# Supplementary figures and images for: Oral Contraceptive Use Influences On-Kinetic Adaptations to Sprint Interval Training in Recreationally-Active Women
Source: Front Physiol. 2020 Jun 12;11:629. doi: 10.3389/fphys.2020.00629 (PMC7303366; doi:10.3389/fphys.2020.00629)

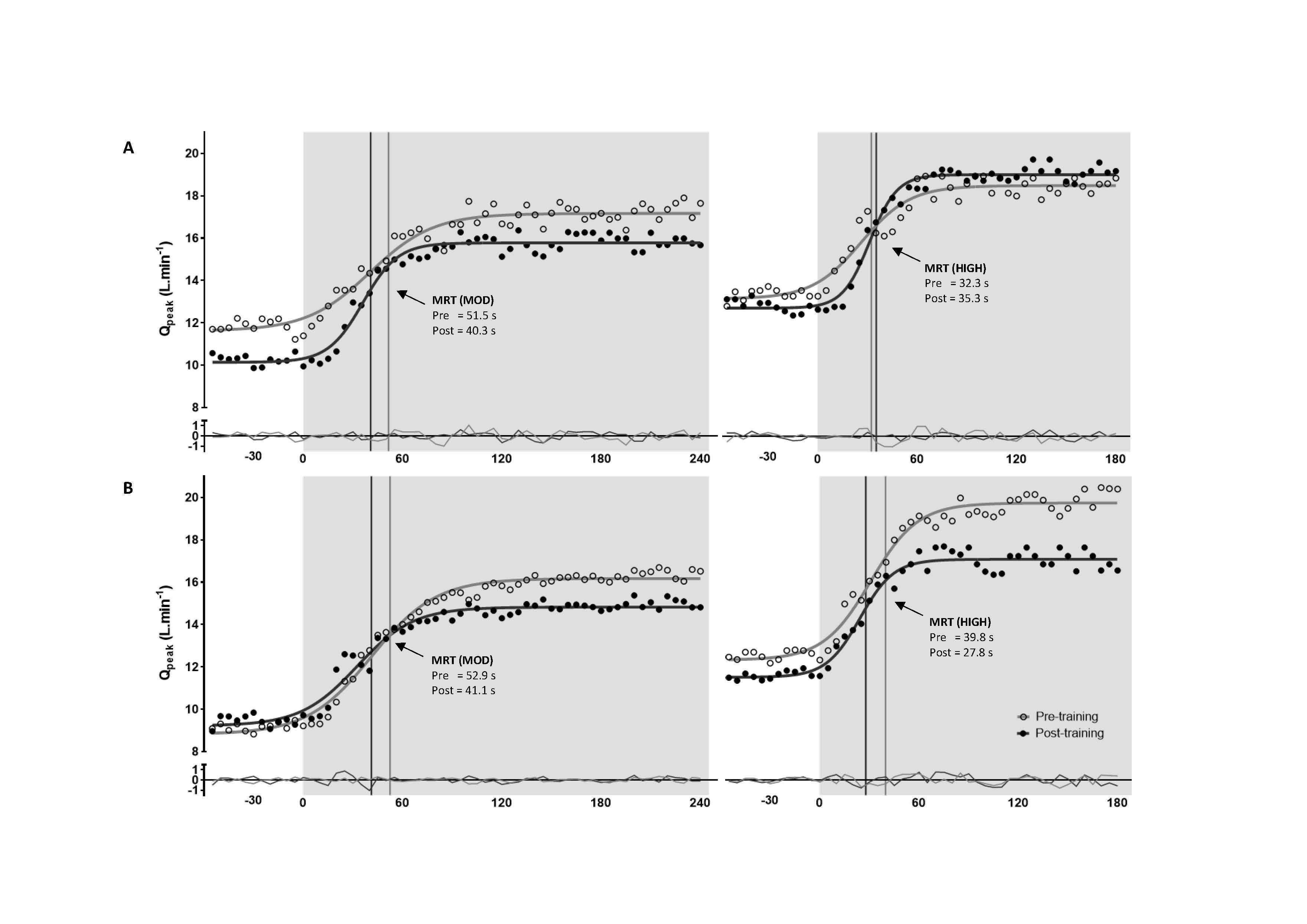

Supplement: FIGURE S1 — Cardiac output profile (with model best fit line and residuals; time in seconds on the x-axis) for a representative naturally menstruating participant (A) and oral contraceptive using participant (B) during the transition to moderate (left) and heavy (right) intensity exercise, pre-training (open circles and gray line) and post-training (closed circles and black line). Mean response time (MRT) is indicated by a matched vertical line. Shading represents exercise onset. [file Image_1.JPEG]

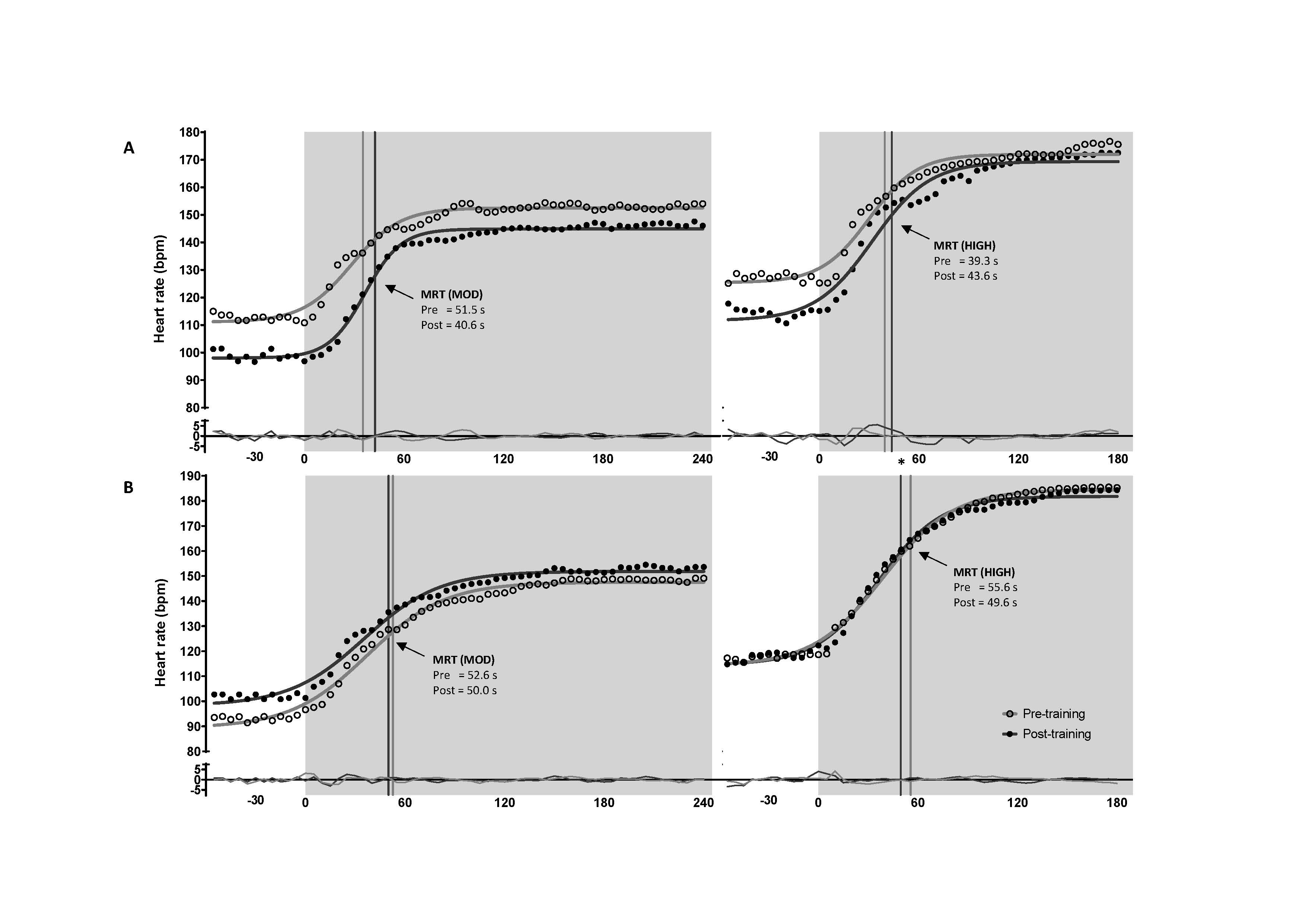

Supplement: FIGURE S2 — Heart rate profile (with model best fit line and residuals) for a representative naturally menstruating participant (A) and oral contraceptive using participant (B) during the transition to moderate (left) and heavy (right) intensity exercise, pre-training (open circles and gray line) and post-training (closed circles and black line). Mean response time (MRT) is indicated by a matched vertical line. Shading represents exercise onset. [file Image_2.JPEG]
