# Supplementary material for: Oral Contraceptive Use Influences On-Kinetic Adaptations to Sprint Interval Training in Recreationally-Active Women
Source: Front Physiol. 2020 Jun 12;11:629. doi: 10.3389/fphys.2020.00629 (PMC7303366; doi:10.3389/fphys.2020.00629)
Supplement: TABLE S1 — Pulmonary oxygen uptake, cardiac output and heart rate on-kinetic responses to moderate and heavy intensity exercise, pre- and post-training in naturally-menstruating women, sub-grouped for normal luteal phase characteristics (n = 16) and apparent luteal phase deficient women (n = 8); as determined by serum progesterone concentrations. [file Table_1.pdf]

Supplementary Material

Supplementary Table 1. Pulmonary oxygen uptake, cardiac output and heart rate on-kinetic responses to moderate and heavy intensity exercise, pre- and post-training in naturally-menstruating women, sub-grouped for normal luteal phase characteristics (n=16) and apparent luteal phase deficient women (n=8); as determined by serum progesterone concentrations.

	**Normal luteal characteristics (n=16)**		**Apparent luteal phase deficient (n=8)**	
	**Pre-training**	**Post-training**	**Pre-training**	**Post-training**
**MODERATE INTENSITY EXERCISE (90% PO_{VT})**				
**Pulmonary oxygen uptake on-kinetic response**				
Baseline $\dot{V}O_2$ ($L.min^{-1}$)	0.7±0.1 (0.6-0.7)	0.7±0.1 (0.6-0.8)	0.8±0.1 (0.6-1.0)	0.6±0.1 (0.4-0.7)*
Amplitude ($L.min^{-1}$)	0.9±0.5 (0.5-1.2)	0.7±0.3 (0.5-0.8)	1.1±0.6 (0.2-2.1)	0.8±0.5 (0.0-1.5)*
Time delay (sec)	22.4±8.7 (16.6-28.2)	23.7±6.9 (19.1-28.4)	26.9±9.1 (12.3-41.4)	32.4±10.1 (16.3-48.5)*
Time constant (*tau*1; sec)	39.5±14.2 (30.0-49.0)	26.4±73.5 (21.3-31.4)*	27.0±11.0 (9.5-44.6)	21.7±8.4 (8.2-35.1)
Mean response time (sec)	61.9±11.1 (54.5-69.4)	50.1±6.3 (45.9-54.3)*	53.9±9.7 (38.4-69.4)	54.1±4.9 (46.2-61.9)
**Cardiac output on-kinetic response**				
Baseline $\dot{Q}$ ($L.min^{-1}$)	10.9±1.5 (10.0-11.7)	10.5±0.9 (10.0-11.0)	11.2±1.8 (9.4-13.1)	10.9±1.2 (9.7-12.1)
Amplitude ($L.min^{-1}$)	4.0±1.3 (3.2-4.7)	4.8±1.6 (3.8-5.7)*	4.1±1.6 (2.4-5.7)	4.3±1.6 (2.7-5.9)
Time delay (sec)	5.8±2.5 (4.4-7.3)	7.6±6.2 (4.0-11.1)	6.4±2.8 (3.4-9.3)	11.7±7.1 (4.3-19.2)*
Time constant (*tau*1; sec)	50.0±11.8 (43.2-56.9)	36.1±10.8 (29.8-42.3)*	49.9±8.6 (40.8-58.9)	30.6±9.6 (20.4-40.7)*
Mean response time (sec)	55.9±11.2 (49.4-62.4)	43.6±6.5 (39.9-47.4)*	56.2±7.3 (48.6-63.9)	42.3±4.2 (37.9-46.7)*
**Heart rate on-kinetic response**				
Baseline heart rate (bpm)	113.5±9.4 (108.1-118.9)	107.1±10.0 (101.3-112.9)*	114.5±6.7 (107.5-121.5)	107.9±10.9 (96.5-119.3)
Amplitude (bpm)	31.98±6.1 (28.4-35.5)	32.4±8.9 (27.3-37.5)	32.1±8.9 (22.7-41.4)	32.4±12.2 (19.6-45.2)
Time delay (sec)	4.6±3.9 (2.3-6.8)	5.9±5.7 (2.6-9.2)	3.4±1.6 (1.7-5.1)	9.2±7.5 (1.3-17.1)
Time constant (*tau*1; sec)	51.9±17.4 (41.9-61.9)	38.4±9.8 (32.8-44.1)*	55.9±11.7 (43.6-68.2)	37.7±12.6 (24.5-50.9)*
Mean response time (sec)	56.5±15.5 (47.5-65.4)	44.3±6.3 (40.7-48.0)*	59.3±10.8 (48.0-70.7)	46.9±5.6 (41.0-52.9)*

**HEAVY INTENSITY EXERCISE (Δ50% PO_{VT})**				
**Pulmonary oxygen uptake on-kinetic response**				
Baseline $\dot{V}O_2$ (L.min^{-1})	0.8±0.1 (0.7-0.8)	0.8±0.1 (1.2-0.3)	0.8±0.1 (0.7-1.0)	0.7±0.1 (0.5-0.9)*
Amplitude (L.min^{-1})	1.2±0.4 (1.0-1.5)	1.2±0.3 (0.9-1.4)*	1.2±0.5 (0.5-2.0)	1.2±0.4 (0.6-1.9)
Time delay (sec)	21.4±6.2 (17.3-25.5)	23.8±5.3 (20.2-27.4)	23.4±7.5 (11.4-35.4)	23.2±1.8 (20.3-26.0)
Time constant *(tau*1; sec)	36.3±6.5 (32.0-40.7)	27.3±8.4 (21.7-33.0)*	28.1±10.0 (12.2-45.9)	25.3±4.2 (18.7-32.0)
Mean response time (sec)	57.7±7.6 (52.6-62.8)	51.1±5.6 (47.4-54.9)*	51.5±11.3 (33.4-69.5)	48.5±5.5 (39.7-57.3)
**Cardiac output on-kinetic response**				
Baseline $\dot{Q}$ (L.min^{-1})	12.7±0.9 (12.2-13.2)	12.1±1.0 (11.5-12.7)	12.8±1.3 (11.5-14.1)	12.5±1.2 (11.3-13.7)
Amplitude (L.min^{-1})	5.6±1.7 (4.7-6.6)	6.4±1.6 (5.5-7.3)*	5.0±1.3 (3.6-6.4)	5.4±1.3 (4.1-6.8)
Time delay (sec)	8.2±3.4 (6.2-10.2)	9.7±4.8 (6.9-12.5)	9.9±4.3 (5.3-14.4)	10.4±5.4 (4.4-16.5)
Time constant *(tau*1; sec)	44.9±14.3 (36.7-53.2)	31.5±9.0 (26.3-36.7)*	43.0±13.9 (28.4-57.6)	26.4±12.4 (13.4-39.3)*
Mean response time (sec)	53.1±14.5 (44.7-61.5)	41.2±5.1 (38.2-44.1)*	52.9±13.0 (39.2-66.5)	36.8±8.2 (28.2-45.4)*
**Heart rate**				
Baseline heart rate (bpm)	124.7±11.2 (118.2-131.1)	120.5±10.4 (114.5-126.5)	128.8±8.8 (119.6-138.1)	121.2±13.3 (107.3-135.2)*
Amplitude (bpm)	50.5±11.5 (43.9-57.2)	49.0±10.1 (43.2-54.9)	45.6±9.2 (36.0-55.2)	46.0±12.0 (33.3-58.6)
Time delay (sec)	10.7±3.0 (9.0-12.5)	6.7±2.1 (5.5-8.0)*	9.2±3.4 (5.6-12.7)	6.9±1.5 (5.4-8.5)
Time constant *(tau*1; sec)	42.6±8.1 (37.9-47.3)	41.6±5.2 (38.6-44.5)	45.2±13.7 (30.9-59.6)	40.7±4.7 (35.7-45.6)
Mean response time (sec)	53.4±8.3 (48.6-58.2)	48.3±5.3 (45.2-51.4)*	54.4±13.7 (40.0-68.8)	47.6±4.6 (42.8-52.4)

PO_{VT}: power output at ventilatory threshold; **sec:** seconds; **$\dot{V}O_2$:** volume of oxygen consumed. **$\dot{Q}$:** cardiac output; **bpm:** beats per minute; **sec:** seconds. Parametric data are presented as mean±SD (95%CI); non-parametric data are presented as median [IQR] (95%CI). *$p<0.05$ vs. pre-training; paired samples t-test.
